# Involvement of low- and middle-income countries in randomized controlled trial publications in oncology

**DOI:** 10.1186/s12992-014-0083-7

**Published:** 2014-12-13

**Authors:** Janice C Wong, Kimberly A Fernandes, Shubarna Amin, Zarnie Lwin, Monika K Krzyzanowska

**Affiliations:** Department of Neurology, Brigham and Women’s Hospital, 75 Francis Street, Boston, MA 2115 USA; Department of Neurology, Massachusetts General Hospital, Harvard Medical School, 75 Francis Street, Boston, MA 2115 USA; Institute for Clinical Evaluative Sciences, G1 44, 2075 Bayview Avenue, Toronto, Ontario M4N 3 M5 Canada; Department of Medicine, University of Toronto, 1 King’s College Circle, Toronto, Ontario M5S 1A8 Canada; Department of Medical Oncology, University of Queensland School of Medicine, Royal Brisbane and Women’s Hospital, Butterfield Street, Herston 4029 Brisbane, Australia; Department of Medical Oncology & Hematology, Princess Margaret Cancer Centre, 610 University Avenue, Suite 5-206, Toronto, Ontario M5G 2 M9 Canada

**Keywords:** Randomized controlled trials, Publications, Cancer, Low and middle income countries, Authorship, Sponsorship

## Abstract

**Background:**

We describe trends in participation by investigators from low- and middle-income countries (LMCs) in publications describing oncology randomized control trials (RCTs) over a decade.

**Methods:**

We used Medline to identify RCTs published in English from 1998 to 2008 evaluating treatment in lung, breast, colorectal, stomach and liver cancers. Data on author affiliations, authorship roles, trial characteristics, funding and interventions were extracted from each article. Countries were stratified as low-, middle- or high-income using World Bank data. Interventions were categorized as requiring basic, limited, enhanced or maximal resources as per the Breast Health Global Initiative classification. Logistic regression was used to identify factors associated with authorship by investigators from LMCs.

**Results:**

454 publications were identified. Proportion of articles with at least one LMC author increased over time from 20% in 1998 to 29% in 2008 (p = 0.01), but almost all LMC authors were from middle-income countries. Proportion of articles with at least one LMC author was higher among articles that explicitly reported recruitment in at least one LMC vs those that did not (76% vs 13%). Among 87 articles (19%) that involved authors from LMCs, 17% had LMC authors as first or corresponding authors, and 67% evaluated interventions requiring enhanced or maximal resources. Factors associated with LMC authorship included industry funding (OR = 3.54, p = 0.0001), placebo comparator arm (OR = 2.57, p = 0.02) and palliative intent treatment (OR = 4.00, p = 0.0003).

**Conclusion:**

An increasing number of publications describing oncology RCTs involve authors from LMC countries but primarily in non-leadership roles in industry-funded trials.

## Introduction

Low- and middle-income countries (LMCs) disproportionately bear the rising global cancer burden [1,2]. Clinical research needs to address this high cancer burden in resource-limited settings, but only 10% of world’s expenditure on health research has focused on issues in lower resource settings [1,3,4]. Simultaneously, clinical trials are becoming increasingly globalized. From 1995 to 2005, trial participation in sites outside of the United States more than doubled, including LMCs [5,6]. A recent study found that 78% of clinical trials evaluating cancer therapies published between 2007 and 2011 were conducted in developed countries, while 22% of trials were conducted in developing countries [7]. However, few studies have quantitatively addressed the role of LMC investigators in global clinical trials or ethical issues associated with their participation [8].

To capture the level of involvement by investigators from different countries in randomized oncology trials, we systematically reviewed a cohort of published trials and describe trends in participation in these publications by LMCs investigators. Trial involvement includes tasks such as enrolling patients (participation) as well as more academic tasks such as participating in writing up study results (authorship). Our primary focus was on authorship trends but we also describe trends in other tasks where the information was available. We focused on oncology randomized control trials (RCTs) over a decade (1998–2008), specifically documenting the role of LMC investigators in clinical trials as reflected in the publication and the applicability of these trials to local resource settings when LMCs participated. We hypothesized that LMC authorship and participation in publications describing oncology RCTs increased over time, but that few LMC authors had leadership roles and few trial interventions were relevant to lower resource settings even when LMC investigators were involved.

## Methods

### Identification of studies

All phase III clinical trials from January 1, 1998 to December 31, 2008 evaluating treatment in five cancer cancers (lung, breast, colorectal, stomach, liver) were systematically identified from MEDLINE. We selected these cancers based on GLOBOCAN 2002 and American Cancer Society statistics which showed these cancers have the highest global mortality rates. Details of the search are listed in the Appendix. Articles retrieved from MEDLINE were then manually screened for inclusion in the study. Inclusion criteria were: 1) pertaining to one of 5 cancer sites (lung, breast, colorectal, liver or stomach), 2) published in English language, 3) evaluating a treatment intervention (chemotherapy, radiation therapy, surgery, palliative care, hormones or monoclonal antibodies/targeted agents), and 4) absence of exclusion criteria. Exclusion criteria were: 1) prevention, screening or diagnostic procedure trials, 2) articles presenting follow-up or updated data from previously published trials, or 3) sub-studies.

### Data abstraction

From each article, information was extracted directly into a pre-designed electronic database, which underwent pilot testing to ensure face validity and inter-observer reliability. The following data were included: 1) year of publication, 2) country affiliations of first, corresponding and middle authors, 3) country affiliations of participating centers and additional investigators (where available), 4) trial characteristics, 5) types of treatment, 6) documentation of ethics, informed consent and funding sources, and 7) whether relevance of study to LMCs was discussed. Based on Gross National Income data available from World Bank statistics (2009), countries were stratified into high ($11,456 or more per capita), middle ($936-$11,455) and low ($935 or less) income groups. Corresponding author was defined as the primary contact author for the publication, while senior author was defined as the last author on the author list. First, corresponding or senior authorship positions were deemed as leadership roles. Participating centers were defined as institutions that were listed in the publication as having enrolled study participants. Interventions were divided into basic, limited, enhanced and maximal categories based on estimated cost and resources required for implementation as established previously for breast cancer and by one of the authors (MKK) for the other cancer sites applying general guiding principles set out by the Breast Health Global Initiative [9]. Briefly, we considered basic resources to be core healthcare services (e.g. modified radical mastectomy); limited resources were key services that required limited finances and infrastructure (e.g. cyclophosphamide, methotrexate, 5-fluorouracil); enhanced resources were optional services that increased the number and quality of therapies (e.g. taxanes); and maximal resources were services that had lower priority in lower resource settings due to high cost or impracticality (e.g. growth factors) [9]. Ethics were reported as documented if the publication explicitly stated that ethics approval was obtained for the study.

### Statistical analysis

Summary statistics were used to present the data. We examined associations between whether studies had any authors from a low or middle income country (yes or no) and the following variables: year of publication, cancer site, funding type, multi- versus single-centre participation, use of placebo, type of treatment, treatment aim, type of randomized trial, ethics documentation, and whether the study mentioned informed consent. Adjusted analyses were performed using logistic regression, entering all variables into the model except for whether the study mentioned informed consent, due to small cell counts. No evidence of multi-collinearity was found (all variables had tolerances > 0.4). Assessment of the model’s calibration with the Hosmer-Lemeshow test (p-value = 0.67) and discrimination (C-statistic = 0.76) did not show lack of fit. Graphics were created in R v 2.13.1 (R Development Core Team, Vienna, Austria) and statistical analyses were conducted using SAS version 9.0 (SAS Institute Inc, Cary, NC).

## Results

### Search results

The MEDLINE search identified 876 publications describing RCTs in lung, breast, colorectal, stomach and liver cancers, of which 454 publications met inclusion criteria. Table 1 summarizes characteristics of these published trials: trials in lung and breast cancer made up the largest proportion of the cohort. Most trials used simple two-arm designs and focused on systemic cancer treatment such as chemotherapy. More than half of the trials evaluated treatment interventions given with palliative intent and 31% explored treatments beyond first line. Approximately 10% used a placebo in the comparator arm. About 42% of trials identified industry as a funding source either exclusively or in combination with other funding sources.

**Table 1:** **Characteristics of the cohort of oncology RCTs included in the analysis**

**Variable**	**Number of papers (N = 454)**	**Percentage**
**Cancer site**		
Lung	177	39.0
Breast	165	36.3
Colorectal	82	18.1
Stomach	29	6.4
Liver	7	1.5
**Trial design**		
Simple two-arm	393	86.6
Multiple arms	48	10.6
Factorial	13	2.9
**Use of placebo**		
Yes	41	9.0
No	413	91.0
**Treatment aim**		
Adjuvant/Curative	140	30.8
Metastatic/Palliative	241	53.1
Both	43	9.5
Supportive measures	30	6.6
**Treatment type**		
Chemotherapy	367	80.8
Surgery	15	3.3
Hormones	40	8.8
Best supportive care	6	1.3
Radiation	66	14.5
Monoclonal antibodies/targeted agents	63	13.9
**First line treatment**		
Yes	309	68.1
No	145	31.9
**Funding source**		
Industry	154	33.9
Mixed	39	8.6
Not for profit	107	23.6
Not specified	154	33.9

**Figure 1:**
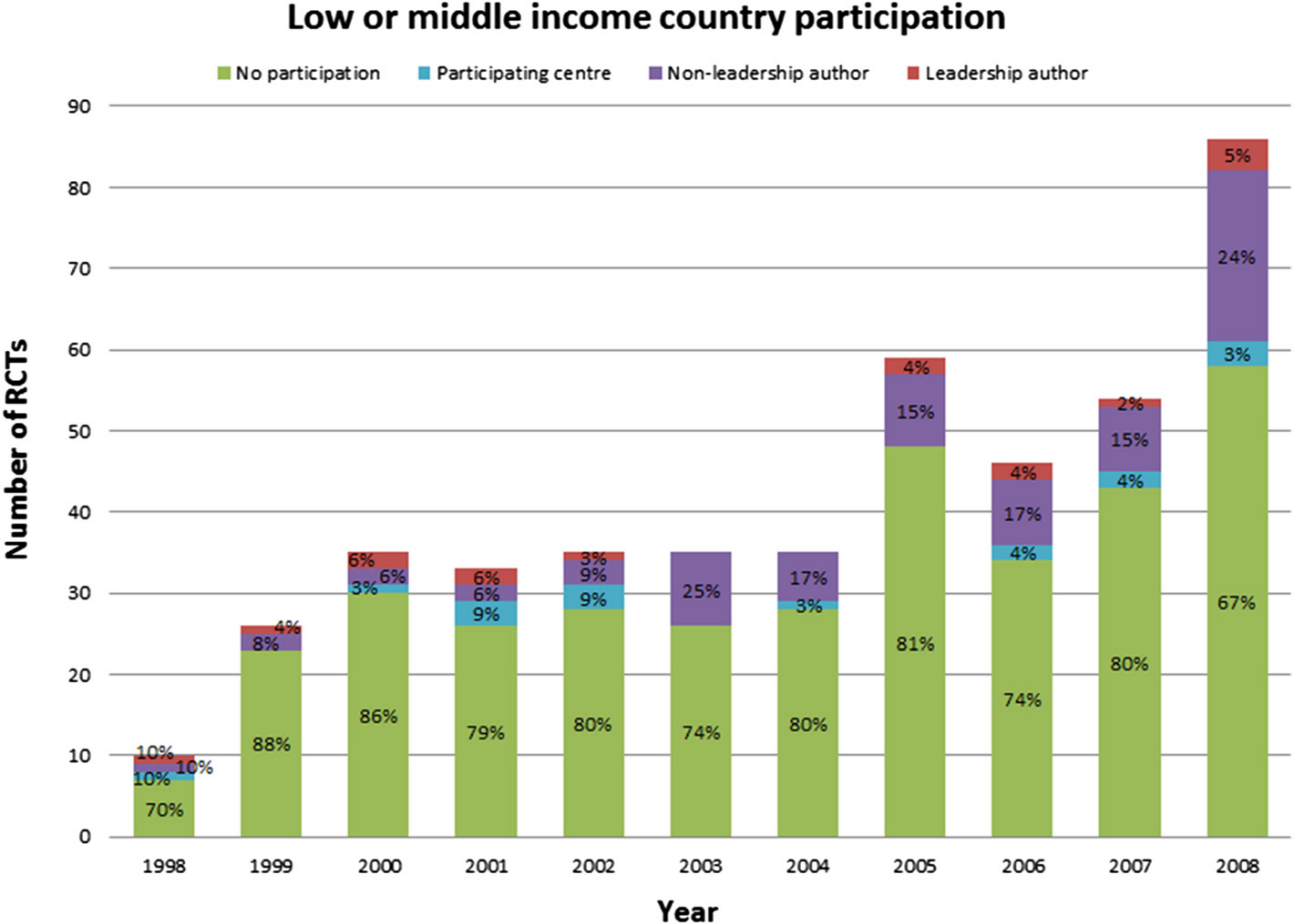
**Temporal trends in low and middle income country participation in oncology RCTs published 1998–2008.**

Figure 1 summarizes temporal trends in the number of publications, stratified by the number of authors or participating centers from LMCs. The number of RCTs published per year increased from 10 in 1998 to 86 in 2008. LMC participation also increased over time from 20% in 1998 to 29% in 2008 (p = 0.01), but most of this increased participation was in non-leadership roles.

### Authors and participating centers

**Figure 2:**
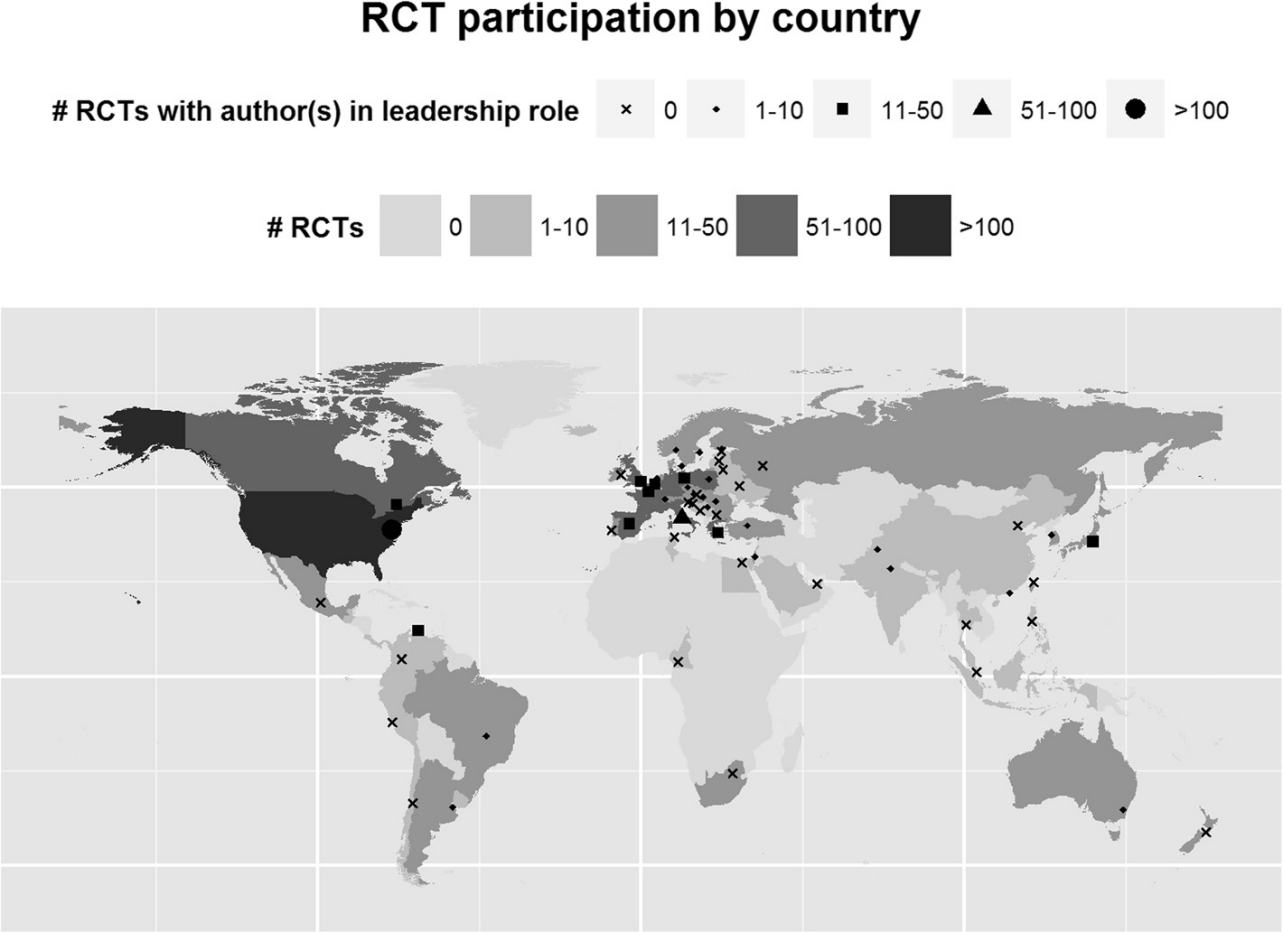
**Map of countries with authors or participating centers in oncology trial publications.** Each country is coded with a color indicating the number of oncology trial publications (light to dark) with a participating center or an author from that country. Each country also has a black symbol (dot) indicating the number of publications with authors from that country in leadership roles.

Figure 2 illustrates the level of participation of each country in RCTs for our study cohort and the number of authors from each country in leadership positions. The median number of authors per publication in our RCT cohort was 12 (range 1–35). Of 454 first authors, 276 could be linked to an academic affiliation or clinical institution, and 440 could be linked to a country. First authors were most commonly from the United States (n = 108), Italy (n = 52) or United Kingdom (n = 34). Corresponding authors were most commonly from the United States (n = 113), Italy (n = 53) or Germany (n = 35). Senior authors were most commonly from the United States (n = 63), Italy (n = 43) or Japan (n = 26). There were no first or corresponding authors from a low-income country, and only two first authors and two corresponding authors from a lower-middle income country (India).

More publications included middle authors from LMCs. Two publications included middle authors from low income countries: Cambodia (n = 1) and Cameroon (n = 1). Multiple publications included middle authors from low-middle income countries: India (n = 7), China (n = 4), Egypt (n = 4), Pakistan (n = 4), Ukraine (n = 3), Philippines (n = 1), Thailand (n = 1) and Tunisia (n = 1).

In addition, we analyzed authorship trends after stratifying by whether the study was conducted in an LMC, high income country or both. The exact number of participating centers was documented in 271 of 454 (60%) publications. The median number of participating centres was 29 (range 1–478). 257 publications (57%) documented the countries involved, so this sub-analysis was limited to this cohort (summarized in Table 2). Centers were most commonly in the United States (n = 61), Italy (n = 58) and Germany (n = 49). Additional investigators who were not authors were acknowledged in 160 publications, and their country affiliations were documented in 154 publications. Ten publications acknowledged participation from LMC centers, but did not have any authors from LMCs. Two studies were conducted exclusively in LMCs; both had at least one author from an LMC, and at least one author from an LMC in a leadership role. Forty studies (9%) were conducted in both LMCs and high income countries; in these studies 30 (75%) had at least one author from an LMC, and 7% had at least one author from an LMC in a leadership role. Two hundred and fifteen studies (47%) occurred in high income countries, with 3 (1%) of these studies including at least one author from an LMC in a non-leadership role.

**Table 2:** **Authorship trends by location of study (n = 454)**

**Where was the study** ***conducted***	**Number of studies (overall)**	**Number of studies with at least one author from LMIC (any role)**	**Number of studies with at least one author from LMIC** ***in a leadership role***
Not explicitly reported	197 (43%)	52 (26%)	10 (19%)
LMIC only	2 (<1%)	2 (100%)	2 (100%)
HIC and LMIC	40 (9%)	30 (75%)	2 (7%)
HIC only	215 (47%)	3 (1%)	0 (0%)
Total	454	87 (19%)	14 (3%)

Using the same stratification by whether the study was conducted in a low and/or high income country, we also analyzed collaboration patterns between low and high income countries for multi-institutional studies (N = 263). Results are presented in Table 3. There was insufficient data for this analysis in 62 studies. Of the remaining 201 studies, one study was conducted in an LMC setting only, with authors from more than one LMC. Thirty four studies were conducted in LMCs and high income countries; 26 of these studies included at least one author from an LMC in addition to authors from high income countries. One hundred and sixty six studies were conducted in high income countries only with the majority of the trials (131, 79%) having authors from only one high income country.

**Table 3:** **Collaboration patterns in multi-institutional studies by location of study (n = 263)**

**Where was the study conducted**	**Number of studies (overall)**	**Number of studies conducted in one country**	**Number of studies conducted in multiple countries**
**Missing**	62	NA	NA
**LMIC only**	1	0	0
All authors from one LMIC	0 (0%)	NA	NA
Authors from >1 LMIC	1 (100%)	NA	NA
**HIC and LMIC**	34	0	34
One HIC and One LMIC author	0 (0%)	NA	0 (0%)
Authors from >1 HIC and >1 LMIC	26 (76%)	NA	26 (76%)
**HIC only**	166	125	41
All authors from one HIC	131 (79%)	119 (95%)	12 (29%)
Authors from >1 HIC	35 (21%)	6 (5%)	29 (71%)
**Total**	263	126	75

NA = not applicable.

### Funding and ethics

**Figure 3:**
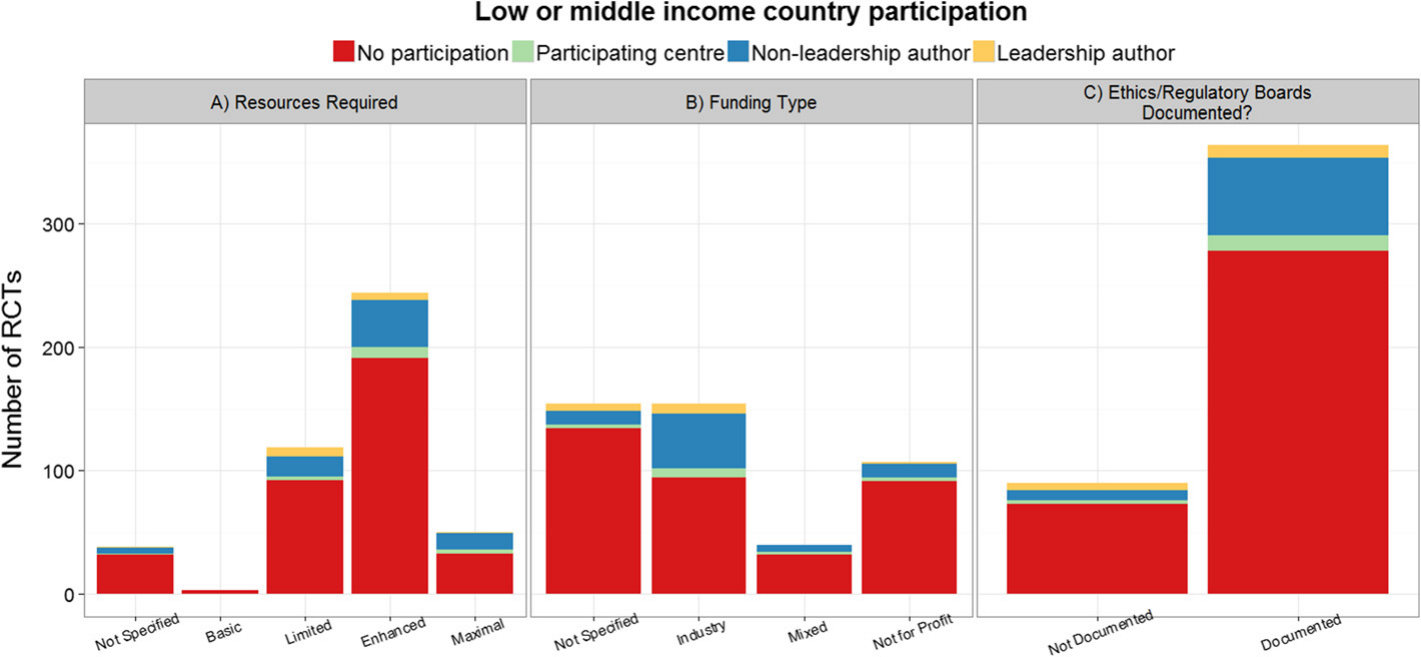
**Funding sources for oncology trial publications.** Graphs show the number of publications with **A)** resource levels required for trial interventions, **B)** types of funding source, **C)** documentation of ethics or regulatory board approval, stratified by the number of authors or participating centers from low- and middle-income countries (LMCs).

Funding sources are summarized in Table 1. Involvement of LMC authors or participating centres stratified by funding source is illustrated in Figure 3. Notably, the proportion of studies involving LMC investigators or centres was highest among purely industry funded trials: 40% of industry-only funded trials had LMC participation, while 15% of trials funded by not-for-profit sources reported LMC participation.

Ethics approval was mentioned in 364 of 454 articles. Informed consent was mentioned in 428 of 454 articles. Only two articles stated that all participants had to speak English fluently. Only 1 article documented that consent forms were translated into a language other than English.

### Relevance to lower resource settings

Figure 3 also shows the number of publications that reported RCTs testing interventions at four resource levels (basic to maximal), broken down by whether LMC authors or participating centers were involved. Almost no studies focused on interventions compatible with basic resources, with majority of trials evaluating interventions that required at least enhanced resources for delivery. Among trials that included LMC investigators, 67% evaluated interventions requiring enhanced or maximal resources. Only 3 studies explicitly mentioned the relevance of treatment under evaluation to lower resource settings in either the discussion or conclusion: these studies briefly mentioned treatment costs in considering treatment, or geographic characteristics that limit medical care.

### Variables associated with LMC authorship

Results of the univariable and multivariable logistic regression models are summarized in Table 4. In univariable analysis, more recent publications (versus earlier publications), industry sponsorship (versus not-for-profit sponsorship), placebo-controlled trials (versus trials without the use of placebo) and trials performed in the metastatic setting (versus trials in the adjuvant setting) were more likely to have at least one author from an LMC. These factors remained significant in multivariable analysis. More recently published trials, placebo-controlled trials, industry funded trials and trials in the metastatic setting were more likely to have an LMC author.

**Table 4:** **Factors associated with LMC authorship in oncology RCTs**

	**Univariate logistic regression**		**Multivariable logistic regression**	
**Variable**	**OR (95% CI)**	**p-value**	**OR (95% CI)**	**p-value**
Year				
*For every 1-year increase*	1.11 (1.02 – 1.20)	0.01	1.12 (1.02 – 1.24)	0.02
Site				
*Lung*	1.00	Referent	1.00	Referent
*Breast*	1.38 (0.82 – 2.33)	0.22	1.34 (0.70 – 2.56)	0.38
*Liver/colorectal/stomach*	0.66 (0.34 – 1.27)	0.21	0.52 (0.24 – 1.10)	0.09
Funding				
*Not for profit/Mixed*	1.00	Referent	1.00	Referent
*Industry*	3.63 (2.00 – 6.58)	<.0001	3.54 (1.85 – 6.78)	0.0001
*Not specified*	0.88 (0.44 – 1.79)	0.73	0.95 (0.45 - 2.03)	0.90
Multi-center				
*No*	1.00	Referent	1.00	Referent
*Yes*	1.24 (0.77 – 2.00)	0.38	0.91 (0.52 – 1.58)	0.74
Placebo-controlled				
*No*	1.00	Referent	1.00	Referent
*Yes*	3.08 (1.57 – 6.07)	0.001	2.57 (1.19 – 5.54)	0.02
Treatment type				
*Basic/Limited*	1.00	Referent	1.00	Referent
*Enhanced/Maximal*	1.11 (0.68 – 1.82)	0.68	0.61 (0.32 – 1.16)	0.13
Treatment aim				
*Adjuvant*	1.00	Referent	1.00	Referent
*Both*	3.67 (1.48 – 9.06)	0.005	5.12 (1.81 – 14.48)	0.002
*Metastatic*	3.38 (1.75 – 6.55)	0.0003	4.00 (1.90 – 8.43)	0.0003
*Supportive measures*	2.67 (0.91 – 7.79)	0.07	1.21 (0.35 – 4.17)	0.76
Design				
*Factorial/multiple arms*	1.00	Referent	1.00	Referent
*Simple 2 arm*	0.96 (0.49 – 1.90)	0.91	0.73 (0.34 – 1.58)	0.43
Ethics				
*Not documented*	1.00	Referent	1.00	Referent
*Documented*	1.36 (0.73 – 2.54)	0.33	0.76 (0.37 – 1.56)	0.45

Outcome variable is having at least one LMC author.

## Discussion

Through this systematic review, we found that the absolute number of publications describing oncology RCTs and the proportion of these publications that involved LMC investigators as authors increased over time. Most of the increased LMC authorship involved investigators from middle- rather than low-income countries and usually in non-leadership roles, ie. as middle authors. There were several publications that listed participating sites in LMCs but did not include investigators from those sites as authors. Since not all the publications listed all participating sites, while the absolute number of publications that included LMC authors has increased over time the proportion of studies that recruit in LMCs and include at least one LMC author may not be changing and may even be actually proportionally decreasing over time. A recent study found that, despite participation in global clinical trials, researchers from lower resource settings had proportionally lower rates of authorship per patient enrollment, compared to researchers from higher resource settings [10]. Our study differs from this previous study by focusing specifically on publications in the area of oncology, and addressing additional issues such as relevance of interventions to LMC settings.

We found that publications describing industry funded RCTs were more likely to have authors from LMCs. This may be related to the spatial scope of the trial as industry funded trials are often larger and multinational thus there may be more opportunity for LMC investigators to participate in the trial. Unfortunately, we were unable to optimally control for spatial scope of the trial as information on participating countries was not available for approximately 40% of the manuscripts. Nevertheless, participation of LMC investigators in industry funded cancer trials can theoretically be mutually beneficial: LMC research centers may reap financial benefits from participating in industry trials, while the pharmaceutical industry may reduce costs in multinational trials by conducting them in LMCs [6,11]. However, ethical concerns have been raised. LMCs centers traditionally have less regulation and transparency in the conduct of research [6,12]. Compared to an average study participant from a higher resource setting, the average LMC study participant more likely perceives financial compensation for trial participation as substantial, or lacks understanding about the concept of clinical trials [6]. The LMC investigators may also be more vulnerable in industry-funded trials: a recent study showed that the discrepancies in authorship between lower and higher resource settings in global clinical trials were exacerbated when the trials were industry-funded [10].

Approximately two thirds of the publications in our cohort that included investigators from LMCs evaluated interventions that required at least enhanced resources for their delivery. This concerning finding resonates with the existing literature on the gap between research efforts and LMC needs. Although LMCs’ involvement in oncology trials may provide them with early access to interventions [5], such interventions may not be feasible or even affordable in low resource health care systems in the real-world setting [6,11]. Furthermore, most cancer drug development efforts do not focus on tumor types most relevant to LMCs: in a review of phase II and III cancer drug trials, treatments for cancers of the breast, lung, prostate and colorectal were most frequently studied [13]. In higher income countries, there was a correlation between the incidence, prevalence and mortality of a type of cancer to the number of trials for that specific cancer [13]. In LMCs, only prevalence correlated, suggesting that trials lacked emphasis on the actual cancers carrying higher mortality in LMCs [13]. Similar disconnect between burden of disease and trials conducted in lower resource settings has been found in other medical conditions [14-16]. However, there are likely also inherent benefits for LMC centers that participate in clinical trials. These include gaining access to newer therapies, other healthcare provisions or improved quality of care during trials [5,11,17], improving infrastructure including equipment or human capital [17], improving global information exchange [5], academic achievement or scientific progress [11].

Global taskforces have discussed potential solutions for the high cancer burden in the lowest resource settings. The World Health Organization has developed global strategies and preventative measures [18]. A number of cancer specific initiatives by oncology organizations have addressed breast cancer in LMCs [19]. For example, the 2007 Breast Health Global Initiative has developed guidelines for radiation therapy [20], pathology [21], treatment resource allocation [22], diagnosis resource allocation [23] and early detection resource allocation [24] in breast cancer. The 2010 Breast Health Global Initiative summit analyzed challenges in breast cancer management in LMCs [25]. The U.S. Centers for Disease Control and Prevention, American Cancer Society and National Cancer Institute are leading other initiatives [19].

How can we improve cancer treatment and outcomes in LMCs? Some authors have noted that improvement in breast cancer survival in the United States occurred before introduction of technological advances, raising the possibility that cancer survival in LMCs may be improved by focusing on increased awareness and early detection of cancers and by optimizing primary care and referral systems [26]. Tele-oncology may also improve cancer survival in LMCs [27]. A focus on primary prevention and screening, early diagnosis, low cost therapy and establishment of regional initiatives may improve care in LMCs [28,29]. Finally, cancer treatment and outcomes are inextricably linked to research, and while improving access to existing treatments is important, we feel that investing in research that is relevant to LMCs will be key to ensure appropriate care relevant to local settings.

Our review has a number of limitations. First of all, given the lag between study design and publication, our review reflected trials that were predominantly designed in the 1990s. A review of trial registries – ideally multiple registries to ensure a comprehensive and international approach – could be helpful to provide a more current overview of LMC involvement in oncology trials. We also did not collect information on when studies were conducted, as studies may have been conducted several years prior to publication. Nevertheless, our review provides a baseline assessment for future comparisons and identifies a number of concerning trends to monitor. Secondly, a number of publications did not provide full listings of participating countries. Underreporting of participating countries probably leads to an underestimation of LMC participation more so than participation from higher resource settings, as higher resource settings were more likely to have been also listed in authorship affiliations. Acknowledgement of LMCs must be more consistent in published manuscripts. Lastly, we have limited our search to English language articles and there is the possibility that LMC relevant trials may have been published in local journals in other languages. We would encourage future studies to look at studies published in other languages.

## Conclusions

An increasing number of articles describing oncology RCTs involved LMC authors but primarily in non-leadership roles. These publications were commonly industry-funded and often reported interventions that required at least enhanced resources for implementation. To minimize concerns of exploitation and expedite global research collaborations, it is crucial that trial interventions are locally feasible and investigators receive appropriate authorship credits.

## Appendix

### Search strategy

- A MEDLINE search was conducted (OVID MEDLINE 1996-June 2009) to identify all published Phase III clinical trials within a 10 year time frame (1998–2008) for the 5 tumour types.
- The search was limited to the English language and humans for the publication years between 1998–2008.
- The search was then limited to include phase III trials only.
- The results of the search were manually reviewed to identify treatment trials.

1. Lung Neoplasms/(56805)
2. limit 1 to (english language and humans and yr = "1998 - 2008") (38278)
3. limit 2 to clinical trial, phase iii (297)
4. Breast Neoplasms/(90306)
5. limit 4 to (english language and humans and yr = "1998 - 2008") (70178)
6. limit 5 to clinical trial, phase iii (345)
7. Stomach Neoplasms/(23934)
8. limit 7 to (english language and humans and yr = "1998 - 2008") (15767)
9. limit 8 to clinical trial, phase iii (37)
10. Colorectal Neoplasms/(28766)
11. limit 10 to (english language and humans and yr = "1998 - 2008") (21816)
12. limit 11 to clinical trial, phase iii (118)
13. Liver Neoplasms/(39339)
14. limit 13 to (english language and humans and yr = "1998 - 2008") (26242)
15. limit 14 to clinical trial, phase iii (59)

## Abbreviations

LMC: Low- and middle-income countries
HIC: High-income country
RCT: Randomized control trial
